# Development and Validation of an SPE–LC–MS Method for the Determination of Epirubicin, Olaparib and Ribociclib in Human Serum

**DOI:** 10.3390/biomedicines14040848

**Published:** 2026-04-08

**Authors:** Monica Denisa Elena Popescu, Costel-Valentin Manda, Octavian Croitoru, Daniela-Maria Calucică, Johny Neamțu, Andrei Biță, Amelia Maria Găman, Simona-Daniela Neamțu

**Affiliations:** 1 Doctoral School, Faculty of Pharmacy, University of Medicine and Pharmacy of Craiova, 2 Petru Rareș Street, 200349 Craiova, Romania; monica.popescu@umfcv.ro; 2 Department of Analytical and Instrumental Chemistry, Faculty of Pharmacy, University of Medicine and Pharmacy of Craiova, 2 Petru Rareș Street, 200349 Craiova, Romania; 3 Department of Drug Analysis, Faculty of Pharmacy, University of Medicine and Pharmacy of Craiova, 2 Petru Rareș Street, 200349 Craiova, Romania; octavian.croitoru@umfcv.ro; 4 Department of General and Inorganic Chemistry, Faculty of Pharmacy, University of Medicine and Pharmacy of Craiova, 2 Petru Rareș Street, 200349 Craiova, Romania; daniela.croitoru@umfcv.ro; 5 Department of Pharmaceutical Physics, Faculty of Pharmacy, University of Medicine and Pharmacy of Craiova, 2 Petru Rareș Street, 200349 Craiova, Romania; johny.neamtu@umfcv.ro; 6 Department of Pharmacognosy, Faculty of Pharmacy, University of Medicine and Pharmacy of Craiova, 2 Petru Rareș Street, 200349 Craiova, Romania; andrei.bita@umfcv.ro; 7 Department of Pathophysiology, Faculty of Medicine, University of Medicine and Pharmacy of Craiova, 2 Petru Rareș Street, 200349 Craiova, Romania; amelia.gaman@umfcv.ro; 8 Department of Immunology and Haematology, Faculty of Pharmacy, University of Medicine and Pharmacy of Craiova, 2 Petru Rareș Street, 200349 Craiova, Romania; simona.neamtu@umfcv.ro

**Keywords:** epirubicin, olaparib, ribociclib, human serum, SPE, LC-MS

## Abstract

**Background/Objectives**: Epirubicin, Olaparib, and Ribociclib are widely used anticancer agents whose serum concentrations exhibit significant inter-individual variability, supporting the need for reliable and robust analytical methods suitable for pharmacokinetic evaluation and therapeutic exposure assessment. Variations in metabolism, drug–drug interactions, organ function, and treatment regimens may substantially influence systemic exposure, highlighting the importance of accurate quantification in clinical practice. This study describes the development and validation of a solid-phase extraction–liquid chromatography–mass spectrometry (SPE–LC–MS) method for the simultaneous quantification of these drugs in human serum. **Methods**: Sample preparation was performed using Oasis PRiME HLB^®^ cartridges to ensure efficient clean-up, optimal recovery, and reduced matrix effects. Chromatographic separation was achieved using gradient elution with 0.1% formic acid and acetonitrile on a reversed-phase column, followed by single-quadrupole mass spectrometric (QDa) detection in the selected ion recording mode. The total run time was 13 min, enabling high-throughput analysis. **Results**: The method demonstrated good linearity (r > 0.997) over the tested concentration ranges, along with adequate selectivity, precision, accuracy, recovery, and stability, fulfilling the ICH M10 guideline validation criteria. No significant carry-over or interference from endogenous compounds was observed. **Conclusions**: Application to patient samples confirmed reliable performance in real clinical matrices and consistent quantification across different concentration levels. The proposed approach provides a potentially more accessible alternative in laboratories already equipped with LC-MS systems compared to LC-MS/MS platforms and can be applied in pharmacokinetic studies, representing a proof-of-concept for exposure assessment in oncology.

## 1. Introduction

Epirubicin (4′-epidoxorubicin) belongs to the class of anthracyclines, which are highly effective drugs in the treatment of various types of cancers in both mono- and combined therapy. Side effects of epirubicin include nausea, hair loss, myelosuppression, anemia and, most importantly, cardiotoxicity [1]. Because of inter-individual variability, age, concomitant diseases and co-administered drugs, standard body-surface-area dosing may generate subtherapeutic exposure or serum toxic effects. The increasing use of combination regimens and targeted therapies in oncology has intensified the need for reliable analytical tools capable of supporting pharmacokinetic evaluation, exposure verification and therapeutic monitoring.

Quantitation methods for epirubicin usually employ high-performance liquid chromatography (HPLC) with fluorescence detection [2,3,4,5], ultraviolet detection [6,7], electrochemical detection [8], and tandem mass spectrometry (MS/MS) [9,10,11]. Although LC-MS/MS is generally considered the reference technique due to its selectivity and sensitivity, its high acquisition and maintenance cost limits its accessibility in many analytical and clinical laboratories.

Olaparib is a potent inhibitor of poly(ADP-ribose) polymerase (PARP), approved by the European Medicines Agency (EMA) in 2014 for the treatment of ovarian cancer [12]. Pharmacokinetic data for this class of drugs reveal substantial inter-subject variability in uptake, metabolism and elimination. Most reported quantification techniques use HPLC-MS/MS [13,14]. However, simpler and more accessible approaches have also been explored; for example, Daumar et al. validated an HPLC method with diode array detection (DAD) for intracellular level estimation of olaparib in cancer cells [15].

The third drug investigated in this study, ribociclib, belongs to the cyclin-dependent kinase (CDK) inhibitor family, which also includes palbociclib, milciclib and abemaciclib. Several HPLC-MS/MS methods have been used in clinical studies, and the results also revealed marked inter-individual differences in plasma/serum concentrations [16,17,18,19,20]. Despite their sensitivity, these approaches are expensive and labour-intensive, and some reports provide only partial validation data, mainly limited to calibration curves [17]. Consequently, there is ongoing debate regarding whether less complex mass spectrometric platforms could provide sufficient analytical performance for routine pharmacokinetic assessment in biological matrices.

While LC-MS/MS methods provide high sensitivity and selectivity, they are associated with several limitations, including high instrumentation and maintenance costs, the need for specialized personnel and limited availability in many routine clinical or academic laboratories. In addition, many published methods focus on single analytes or lack full validation in complex biological matrices, which restricts their applicability to multi-drug analysis in real clinical settings.

Alternative approaches such as HPLC with UV, fluorescence or electrochemical detection are more accessible but often suffer from lower selectivity, higher susceptibility to matrix interference and insufficient sensitivity for low concentration levels [2,3,4,5,6,7,8]. As a result, there is a need for intermediate analytical strategies that balance performance with accessibility, particularly in laboratories where LC-MS/MS instrumentation is not readily available [9,10,11,13,14,15].

Although epirubicin, olaparib and ribociclib belong to different therapeutic classes (anthracyclines, PARP inhibitors and CDK4/6 inhibitors, respectively), they are increasingly relevant in modern oncology either as part of combination regimens or as sequential therapeutic strategies. The selection of these three compounds was motivated by their clinical importance, their distinct physicochemical properties, and the analytical challenge associated with their simultaneous determination in a complex biological matrix such as human serum.

Moreover, all three drugs exhibit significant inter-individual pharmacokinetic variability [16,17,18,19,20,21,22,23,24,25,26,27,28], which supports the need for reliable analytical methods applicable to pharmacokinetic studies and exposure assessment. Developing a single analytical platform capable of quantifying compounds with different chemical characteristics may improve laboratory efficiency and facilitate broader access to drug monitoring in clinical and research settings.

Previous studies have demonstrated that single-quadrupole mass spectrometric systems operated in selected ion recording (SIR) mode can provide adequate sensitivity and selectivity for the quantitative analysis of drugs in biological matrices when chromatographic separation is optimized and analyte concentrations are within the ng/mL–µg/mL range [9,10,11,16,17,18,19,20].

Furthermore, several validated LC-MS/MS methods for anticancer drugs report similar ranges of linearity, precision, accuracy and LLOQ to those achieved in the present study [9,10,11,20], suggesting that comparable quantitative performance can be obtained for pharmacokinetic and exposure assessment purposes.

In addition, solid-phase extraction using hydrophilic–lipophilic-balanced sorbents has been consistently reported to reduce matrix effects and improve method robustness in serum analysis [2,4,5].

In this context, we hypothesized that a single-quadrupole mass spectrometry (QDa) platform combined with optimized solid-phase extraction (SPE) clean-up could provide an appropriate balance between analytical performance and accessibility for the simultaneous quantitation of epirubicin, olaparib and ribociclib in human serum.

Our research therefore aimed to develop and validate an HPLC-MS method applicable to pharmacokinetic sampling, exposure verification and adherence assessment. Oasis PRiME HLB^®^ cartridges were used for serum sample preparation. Proteins and phospholipids can generate significant analytical problems when large quantities are introduced into LC systems; therefore, optimization of the clean-up procedure was an important objective to reduce potential interferences at the LC-MS interface. Mass spectrometric conditions were optimized to minimize background noise and ensure clear chromatographic signals. The method was validated according to the International Council for Harmonisation (ICH) M10 guideline on bioanalytical method validation and study sample analysis. The developed approach was further evaluated on real patient samples, demonstrating practical applicability in clinical matrices and suggesting its potential as an accessible alternative to LC-MS/MS-based assays.

## 2. Materials and Methods

### 2.1. Reagents

Epirubicin hydrochloride (98% HPLC, Cat. No. 44583, Lot No. 0000458098) and olaparib base (98% HPLC, Cat. No. SML3705, Lot No. 0000451422) were provided by Sigma-Aldrich (Taufkirchen, Germany). Ribociclib base (99.99% HPLC, Cat. No. S7440, Lot No. S744004) came from Selleck Chemicals (Houston, TX, USA). Purity was considered as received in all cases, without correction for potency. Formic acid (98–100%, Cat. No. 1.11670.1000, Lot No. B0203270802) and LC-MS analytical grade solvents, acetonitrile (gradient grade > 99.9%, Cat. No. 34851, Lot No. STBG3844V), water (LC-MS grade, Cat. No. 1.1533.2500, Lot No. Z0880233 327), and methanol (> 99.9%, Cat. No. 34885, Lot No. SZBG106EV) were purchased from Merck (Bucharest, Romania). Oasis PRiME HLB^®^ SPE cartridges (1 cc/30 mg, Cat. No. 186008055, Lot No. 021034248A) were provided by Waters (Bucharest, Romania).

Blood Transfusion Centre (Craiova, Romania) supplied blank human serum samples used in method validation procedures. Serum was collected from healthy, drug-free donors. Samples from six different individuals were evaluated, which is particularly important for selectivity and matrix effect evaluation. Serum was obtained after blood clotting and centrifugation, then stored at −20 °C until analysis.

### 2.2. HPLC-MS System and Working Conditions

Chromatographic analysis used a Waters (Milford, CT, USA) Arc System, and detection was performed via coupling with an ACQUITY QDa mass detector (Waters, Milford, CT, USA). A Waters CORTECS C18 analytical column, 4.6 × 50 mm, 2.7 μm particle size (Cat. No. 186007375, Lot No. 0158390281) was employed for the sample components’ separation. Two solvent tanks were prepared: solvent A, containing acetonitrile with 0.1% formic acid, and solvent B, containing 0.1% formic acid aqueous solution. Formic acid solutions were prepared by adding 1 mL of formic acid and 999 mL (up to 1 L) of water or acetonitrile (HPLC grade). A 0.22 μm nylon membrane filter was used to remove particles and prevent pump, column, or detector problems. Degassing was performed for 15 min in an ultrasonic bath. A gradient elution was performed. From 0 to 1 min, the ratio was 10% solvent A and 90% solvent B; then, between 1 and 10 min, eluent A was increased to 50%, and between 10 and 12 min it was increased to 70%. Solvent A was decreased back to 10% between 12 and 13 min. Column volume (because of dimensions and porosity) was about 0.5 mL, so the re-equilibration of the stationary phase was fast (5 min). The most important parameter for system suitability criteria per run is the back pressure in the chromatographic column, which must reach the value from the start of the analytical run. A 4 min delay between consecutive analytical runs was set in the instrument software to resolve this problem. The temperature for the column compartment was adjusted to 30 °C. The final re-dissolved extracts were placed in the autosampler and kept at 20 °C while waiting for the analysis. The best mobile phase flow rate was found to be 0.6 mL/min. A 5 μL aliquot was injected into the LC column.

The single-quadrupole mass spectrometer (QDa) mass detector mechanism is based on the electrospray ionization (ESI) of molecules. Parameter adjustment included capillary voltage, which was set to 0.8 kV, and cone voltage, with a value of 25 V. Mass spectra were recorded in positive ion mode (ESI+) in the 100–600 *m*/*z* range for epirubicin and 100–500 for olaparib and ribociclib. Quantitative analysis was assessed in selected ion recording (SIR) mode using protonated molecular ions at *m*/*z* 544 in the case of epirubicin and *m*/*z* 435 for the other two compounds. Method settings and data processing were controlled using EmPower 3 software.

### 2.3. Stock and Working Solutions

Methanol was used as a solvent for preparing all standard solutions. Separate stock solutions for each analyte were prepared, by weighing appropriate amounts of standard substances (epirubicin as hydrochloride salt, olaparib, and ribociclib as base forms), to achieve a concentration of 1 mg/mL (base form). No pH adjustment was performed for dissolution. Stock solutions were mixed to obtain adequate working solutions by dilution with methanol, to obtain final solutions to be added to blank serum, and to perform validation data. Each calibration curve (CC)-level solution contains a mix of the three analytes in the following concentrations: 0.05, 0.50, 2, 5, and 10 µg/mL for epirubicin; 0.10, 1, 10, 20, and 50 µg/mL for olaparib; 1, 2.50, 20, 50, and 100 µg/mL for ribociclib. The solution for the first CC level, corresponding to each compound at the lowest limit of quantitation (LLOQ), contains epirubicin, olaparib, and ribociclib at 0.05, 0.10, and 1 µg/mL concentrations, respectively. The next four CC levels have the following concentrations of each analyte: 0.50, 1, 2.50 µg/mL (second level), 2, 10, 20 µg/mL (third level), 5, 20, 50 µg/mL (fourth level) and 10, 50, 100 µg/mL (fifth level) for epirubicin, olaparib and ribociclib, respectively. All working solutions above 5 µg/mL are prepared by pipetting appropriate volumes of stock solutions, without intermediate concentrations. For example, the fifth concentration level is prepared by adding 10 µL epirubicin stock solution (containing 10 µg epirubicin), 50 µL olaparib stock solution (containing 50 µg olaparib), and 100 µL ribociclib stock solution (containing 100 µg ribociclib) into a small vial, then filling up the volume to 1 mL with methanol (adding 840 µL). For levels lower than 5 µg/mL, the lowest concentrations prepared as presented above are used: 5 µg/mL epirubicin solution (used to prepare by dilution 0.05, 0.50, and 2 µg/mL levels), 10 µg/mL olaparib solution (used to prepare by dilution 0.10 and 1 µg/mL levels), and 20 µg/mL ribociclib solution (used to prepare by dilution 1 and 2.5 µg/mL levels).

Standard solutions were re-prepared every 30 days, when necessary.

### 2.4. Sample Preparation

First, a volume of 10 µL mixed working solution (concentrations presented above) was evaporated with nitrogen in a 2 mL conical ampoule to eliminate methanol, and the residue was diluted in 100 µL blank serum. Then, the sample obtained was added to Oasis PRiME HLB SPE cartridges (Waters, Milford, CT, USA) using overpressure, manually, with a syringe. A 500 µL aqueous solution with 5% methanol was used in the washing step. The elution step consisted of 500 µL of methanol. The three steps were completed with a flow rate of approximately 1 drop/second. The sample was directly loaded into the cartridge without pre-treatment. The 5% methanol washing solution was prepared daily by mixing methanol and water, 5:95 (*v*/*v*) (both HPLC grade). The resulting solution was evaporated to dryness in a nitrogen stream, and an aliquot of 50 µL solution consisting of the initial composition of the mobile phase: solvent A/solvent B, 10:90 (*v*/*v*) was added. The solution was vortexed for 2 min and sonicated in an ultrasonic water bath for 5 min to achieve complete solubilization. The reconstituted solution was placed in the autosampler. A total of 5 µL was injected into the chromatographic column.

### 2.5. Validation Data

Validation parameters were assessed according to the International Council for Harmonisation (ICH) M10 guideline on bioanalytical method validation.

LLOQ was defined as the lowest analyte concentration that could be quantified with acceptable precision (%RSD ≤ 20%) and accuracy (within ±20%), and a signal-to-noise ratio of at least 10:1.

Selectivity was evaluated using six individual non-lipemic and non-hemolyzed human serum sources by comparing responses from blank matrices with those obtained from serum samples spiked at the LLOQ level. Interfering peak areas did not exceed 20% of the analyte response at the LLOQ.

Matrix effects, mainly caused by co-eluting endogenous compounds that may induce ion suppression or enhancement, were evaluated using the standard three-set approach. Three sets of samples were prepared: neat standard solutions in methanol, post-extraction spiked serum samples, and pre-extraction spiked serum samples. Matrix factors were calculated as the ratio of the analyte peak area in post-extraction spiked samples to that in neat standards. Extraction recovery was determined from pre-extraction spiked samples and expressed as a percentage relative to neat standards. Consistent recoveries and acceptable variability across concentration levels were considered indicative of reliable extraction efficiency. The relative standard deviation (%RSD) of matrix factors and recoveries was determined across multiple serum lots to confirm method reproducibility in biological matrices. Three replicates at all calibration curve (CC) levels obtained from five different serum sources were analyzed, and precision expressed as %RSD was required to be <15%.

Calibration curves were constructed by plotting peak area versus analyte concentration using linear regression with 1/x weighting. Five calibration levels were prepared and each level was injected five times. An analytical batch was accepted if at least 75% of non-zero calibration levels, including the LLOQ, were within ±15% of the nominal concentrations (±20% at the LLOQ).

The stability of the analytes in serum was evaluated according to International Council of Harmonisation (ICH) recommendations. Long-term stability was assessed after storage at −20 °C for 30 days (*n* = 5). Freeze–thaw stability was evaluated over three freeze–thaw cycles (*n* = 5). Bench-top stability at 20 °C was assessed for 12 h (*n* = 5) to investigate potential degradation during sample preparation or residence in the autosampler prior to injection.

Accuracy and precision were evaluated at all five CC levels of each analyte. Acceptance criteria were ±15% for both accuracy and precision at all concentration levels and ±20% at the LLOQ. Inter-day precision and accuracy were evaluated over five independent analytical runs performed on three different days by two operators. To minimize bias, working solutions were prepared from independent stock solutions.

Carry-over was evaluated by injecting a blank serum sample after a sample containing analytes at the highest calibration level. Peak areas in blank samples did not exceed 20% of the LLOQ response. To minimize carry-over effects, the autosampler needle was washed ten times with the initial mobile phase composition following injections of high-concentration samples.

### 2.6. Method Application on Real Samples

The study was conducted in accordance with the Declaration of Helsinki and approved by the institutional ethics committee. Patients provided written informed consent for the acquisition of blood. The method was applied to real serum samples; epirubicin blood samples were collected 2 h post-dose from three patients treated with 145 mg epirubicin (Epirubicin Teva, 2 mg/mL); samples for olaparib were collected 10 h post-administration of a 300 mg dose from three patients undergoing treatment with Lynparza^®^ 150 mg; ribociclib was determined in the serum obtained from one patient treated with a 600 mg dose, two hours after drug administration (Kisqali^®^ 200 mg). Sampling time was selected based on the expected pharmacokinetic profile of drugs. Dose administration time was verified from clinical records. In all cases, 2 mL venous blood was collected into Vacutainer^®^ tubes (BD, Franklin Lakes, NJ, USA). Blood samples were allowed to clot at room temperature for 30 min and were centrifuged within 45 min of collection at 1800× *g* for 10 min. Dose timing was verified from patients’ records, ensuring that the reported post-dose sampling times were accurate. When serum was not immediately analyzed, samples were frozen at −20 °C. Before analysis, frozen samples were thawed at 20 °C and extracted according to the sample preparation procedure.

## 3. Results

### 3.1. Method Validation Results

#### 3.1.1. Chromatograms

**Figure 1:**
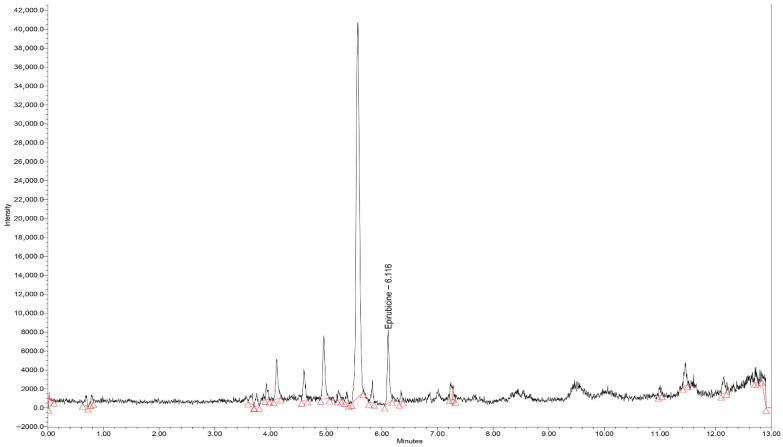
Chromatogram (SIR mode—*m*/*z* 544) for the extract of epirubicin standard serum (50 ng/mL). Red triangles indicate the retention times of the analytes.

**Figure 2:**
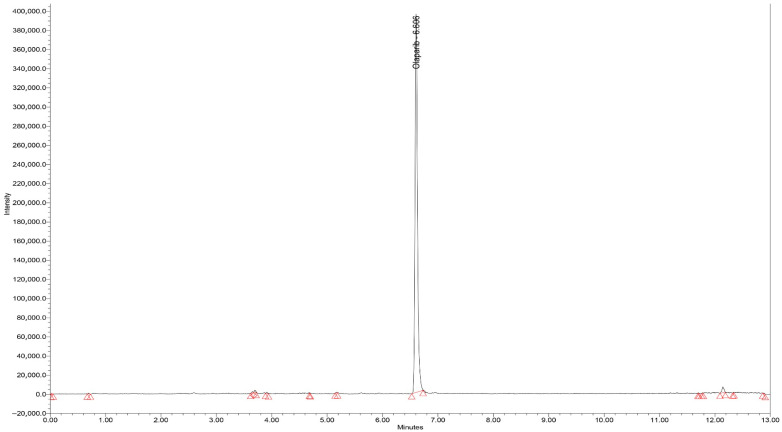
Chromatogram (SIR mode—*m*/*z* 435) for the extract of spiked olaparib standard serum (1000 ng/mL).

**Figure 3:**
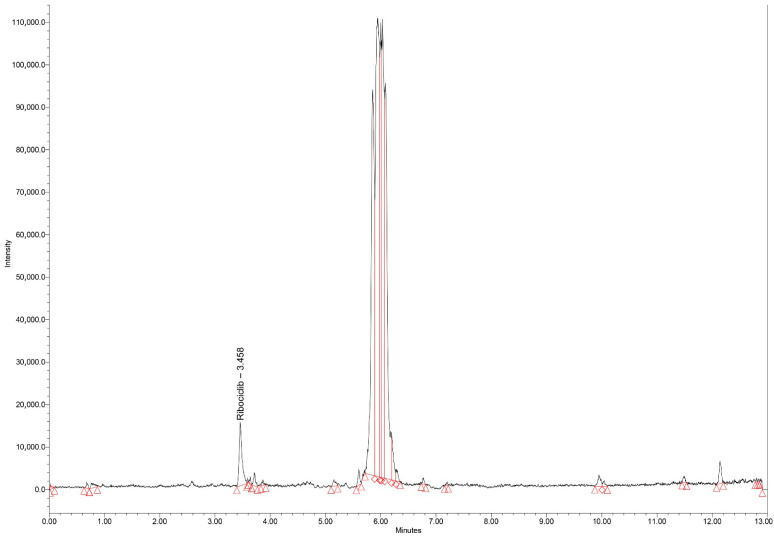
Chromatogram (SIR mode—*m*/*z* 435) for the extract of spiked ribociclib standard serum (2000 ng/mL). Red triangles indicate signals detected at the monitored *m*/*z* values that do not correspond to the analytes and do not interfere with their retention times.

Typical chromatograms of standard samples in SIR mode, for all analytes, with elution times of 6.11, 6.60, and 3.45 min for epirubicin, olaparib, and ribociclib, respectively, are presented in Figure 1, Figure 2 and Figure 3. All peaks were identified by spiking the extracts with corresponding standards before final injection in the analytical column.

**Figure 4:**
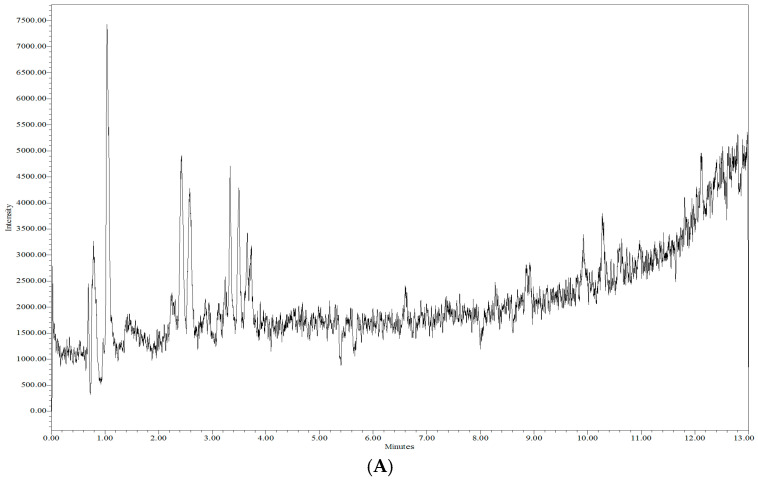

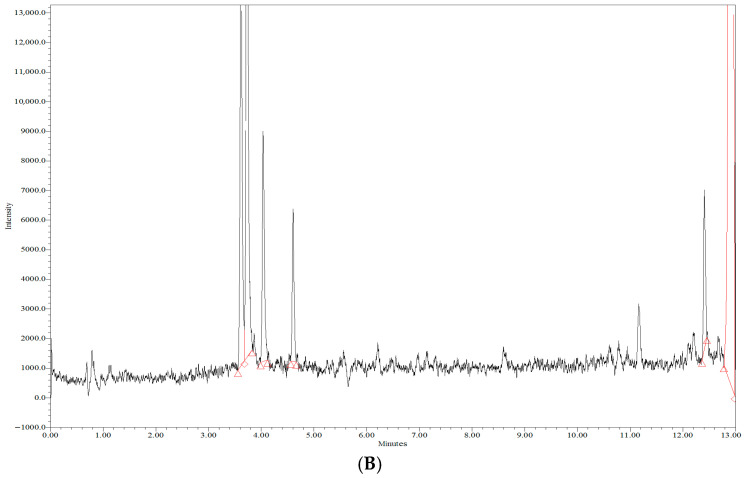
Blank serum chromatograms (SIR mode—*m*/*z* 435 (**A**) and SIR mode—*m*/*z* 544 (**B**)) showing the absence of interfering peaks at analyte retention times.

Blank serum chromatograms presented in Figure 4 showed minor signals at the monitored *m*/*z* values at retention times distinct from those of the analytes. These signals are attributed to endogenous components varying between serum sources. As no co-eluting peaks were observed at the analyte retention times, no interference with quantification was detected.

#### 3.1.2. Selectivity

Selectivity was also investigated. After the evaluation of six individual sources of serum, the peak areas of blank samples were compared with the peak areas of spiked samples at the LLOQ levels of the analytes. No significant increase attributable to unknown compounds in serum was observed. Interference was less than 7% at the LLOQ of each analyte at the specified retention times and *m*/*z* values (544 and 435), which is well below the acceptance criterion of 20% defined in the ICH M10 guideline.

Although olaparib and ribociclib share the same molecular weight, their chromatographic separation was sufficient to provide distinct retention times (elution times of 6.60 and 3.45 min, respectively), preventing co-elution. The MS detector monitored the molecular ion at each retention time, ensuring the selective detection of each compound. The absence of interfering signals at these retention times in blank serum samples further supports the selectivity of the method. Therefore, analyte identification was based on the combined criteria of retention time, chromatographic resolution, and selective ion monitoring.

#### 3.1.3. Matrix Effect

An evaluation of matrix effects, as presented in Section 2, revealed precision below 15% for all calibration curve (CC) levels, as defined in Section 3.1.4, across different individual serum sources. These results demonstrate reproducibility of the method in variable biological matrices, based on an analysis of five independent serum samples.

However, full inter-individual validation of the precision and accuracy at QC levels was not performed and represents a limitation of the study. A more extensive evaluation including a larger number of individual matrices would further strengthen the method validation.

#### 3.1.4. Calibration Curves

**Figure 5:**
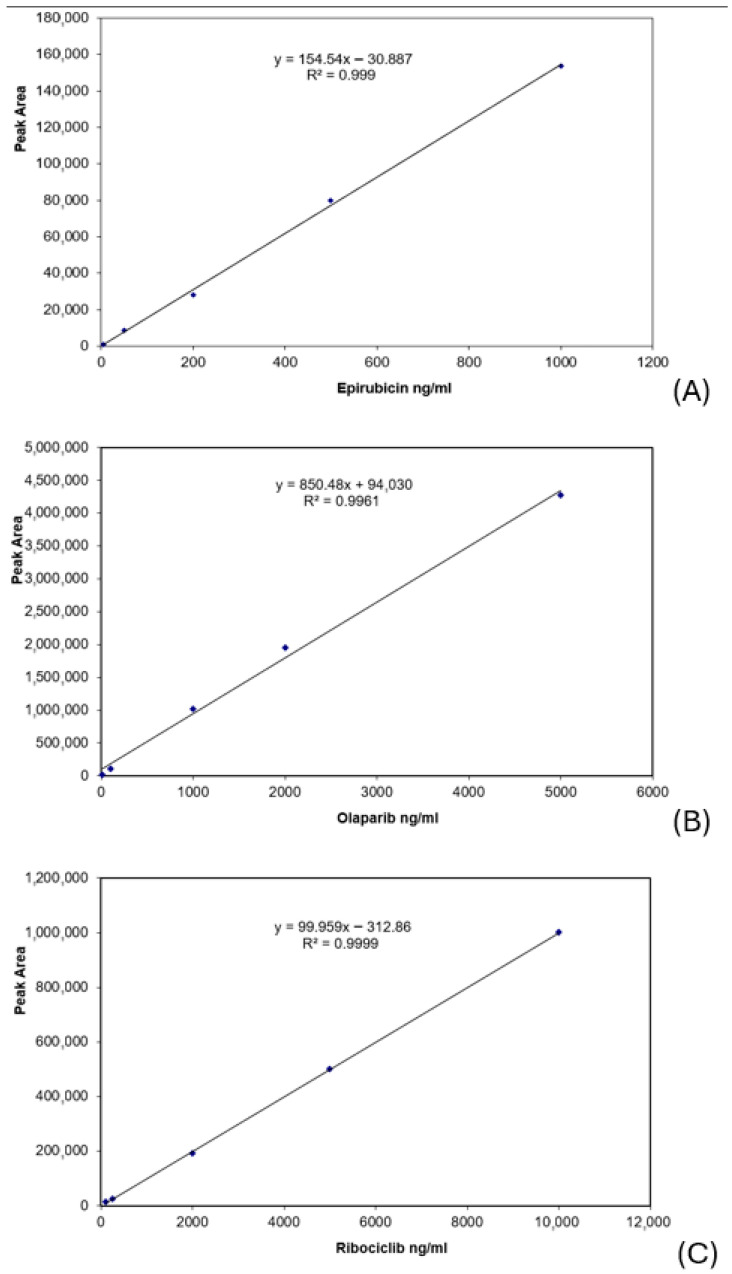
Calibration curves, linear regression equations, and correlation coefficients for epirubicin (**A**), olaparib (**B**), and ribociclib (**C**).

Ranges were selected based on previously reported pharmacokinetic data for the investigated drugs, ensuring coverage of clinically relevant concentration levels [23,24,25,26,27,28,29,30,31,32]. The results for CCs are presented in Figure 5. The ordinate axis indicates detector response, represented by the peak area of each analyte; the X axis presents the nominal serum concentration level. Regression equations with correlation coefficients show the linear response in the selected concentration range.

The LLOQ was determined based on precision, accuracy, and signal-to-noise ratio, ensuring robustness. Values were 5, 10, and 100 ng/mL for epirubicin, olaparib, and ribociclib, respectively, and were ultimately determined based on the signal-to-noise ratio of 10:1 for each compound, as precision and accuracy were consistently well within the ±20% acceptance criteria.

#### 3.1.5. Extraction Recovery and “Carry-Over”

Extraction recovery values were consistently higher than 84% for all analytes across all concentration levels investigated, demonstrating efficient analyte extraction from the serum matrix. The variability of recovery between concentration levels and serum sources remained within acceptable limits, with relative standard deviation values below 15% (RSD < 15%), in accordance with commonly accepted bioanalytical validation criteria.

Carry-over was evaluated by injecting blank samples after the highest calibration level, and the observed responses were below 10% of the LLOQ signal for all analytes, indicating negligible sample-to-sample contamination under the applied conditions.

#### 3.1.6. Stability Test

Stability studies demonstrated that all analytes remained stable under the tested conditions, including long-term storage at −20 °C, three freeze–thaw cycles, and short-term exposure at room temperature (Table 1). Measured concentrations ranged between 95 and 100% of nominal values for all analytes and concentration levels.

The low variability observed (RSD < 15%) confirms that no significant degradation occurred during sample handling, storage, or preparation, supporting the reliability of the method for routine analysis of biological samples.

#### 3.1.7. Precision and Accuracy

Intra- and inter-day precision and accuracy were evaluated at all calibration levels for each analyte (Table 2). Precision values (expressed as %RSD) ranged between 4.32% and 8.56%, while accuracy ranged from 95.0% to 109.20%.

All values complied with the acceptance criteria defined by the ICH M10 guideline, namely, ±15% for precision and accuracy at all concentration levels and ±20% at the LLOQ. In particular, the inter-day accuracy for epirubicin at the LLOQ level (109.20%) remains within the acceptable range (80–120%), confirming the method’s validity at low concentration levels.

System suitability and method robustness were supported by consistent retention times (variation < 2%) and reproducible peak areas across repeated injections, indicating stable chromatographic and detection conditions.

Overall, the validation results demonstrate that the proposed SPE–LC–MS method provides adequate selectivity, precision, accuracy, and robustness for the quantification of epirubicin, olaparib, and ribociclib in human serum within the tested concentration ranges.

### 3.2. Concentration Levels in Real Serum Samples

The linear regression equations presented in Figure 5 were used to calculate the concentration levels of epirubicin, olaparib, and ribociclib in real serum samples. The calibration ranges were selected to encompass the expected clinical concentrations and were confirmed by the analysis of patient samples. Epirubicin levels at 2 h post-dose for three different patients were 124.70, 210.50, and 220.13 ng/mL, respectively. For the other three patients, calculated values for olaparib serum levels at 10 h post-dose were 1322.91, 2991.34, and 2764.20 ng/mL. The only sample available for ribociclib revealed a concentration of 547.13 ng/mL, 2 h post-dose. These concentration levels are consistent with previously reported pharmacokinetic data. Epirubicin concentrations measured 2 h post-dose correspond with the expected post-distribution phase [29]. Olaparib levels at 10 h post-dose fall within the reported steady-state concentration range [30,31], while ribociclib concentrations are consistent with known exposure levels during clinical treatment [25,26,27,28,32].

A direct experimental comparison with LC-MS/MS methods was not performed in this study. However, the obtained validation parameters (linearity, precision, accuracy and LLOQ) fall within ranges reported for LC-MS/MS assays for similar anticancer drugs in biological matrices [9,10,11,16,20]. These findings suggest that the proposed method provides a comparable quantitative performance for the intended application, particularly for pharmacokinetic sampling and exposure assessment, where the analyte concentrations are well above the LLOQ.

This analysis of patient samples should be considered as a proof-of-concept demonstration of the method’s applicability in real biological matrices based on a limited number of samples and single time-point measurements, and not as a full pharmacokinetic or exposure assessment study.

## 4. Discussion

Although no internal standard was used, the method was fully validated for precision, accuracy, and extraction recovery at all CC levels, including the LLOQ. Inter- and intra-day precision and accuracy were consistently within ±15% (±20% at LLOQ), demonstrating reliable quantification in real samples. Strictly controlled sample handling and standardized extraction procedures minimized variability that would otherwise require correction with an internal standard. However, the absence of an internal standard may increase susceptibility to variability arising from matrix effects and instrumental fluctuations, which should be considered when interpreting quantitative results. Slight deviations observed at the LLOQ level, such as the inter-day accuracy of 109.20% for epirubicin, are expected due to increased variability at low concentration levels and do not affect method validity, as they remain within the ±20% acceptance criteria defined by regulatory guidelines.

The absence of an internal standard prevents the calculation of IS-normalized matrix factors. However, consistent precision and accuracy across all CC levels and serum lots indicate that the matrix effects are controlled at the method level. For this reason, calibration curve (CC) samples were used instead of conventional QC levels (LQC, MQC, HQC).

Matrix effects are a known limitation of electrospray ionization bioanalysis, leading to ion suppression or enhancement [33,34,35,36]. However, an acceptable quantitative performance can be achieved when matrix variability is minimized through sample preparation and chromatographic separation [36,37,38]. In the present method, solid-phase extraction using Oasis PRiME^®^ HLB cartridges removed a significant fraction of phospholipids and proteins, reducing matrix interference. The absence of interfering peaks at analyte retention times and the <15% variability observed across independent serum sources demonstrate that residual matrix effects are reproducible rather than random. Under matrix-matched calibration conditions, such effects do not compromise quantitative reliability [35,38].

Sample preparation by SPE using Oasis PRiME^®^ HLB cartridges was selected as a suitable approach based on its reported efficiency and compatibility with biological matrices. The simplified “three-step clean-up” protocol reduces solvent consumption and analysis time. In our conditions, acetonitrile was not suitable as an elution solvent due to its low analyte solubility, particularly for ribociclib, resulting in reduced extraction recovery (~40%) whereas methanol provided significantly improved recovery. Chromatographic separation was achieved using a CORTECS C18 column with gradient elution of acetonitrile and 0.1% formic acid, ensuring the adequate resolution of analytes from matrix components.

SIR detection on a single-quadrupole mass spectrometer (QDa) was selected as a compromise between selectivity, sensitivity, and accessibility. Although it does not provide structural confirmation comparable to MS/MS, sufficient chromatographic separation minimized the risk of co-elution and ensured reliable quantification.

While tandem mass spectrometry is considered the gold standard, single-quadrupole instruments can provide adequate quantitative performance when analyte concentrations are above the LLOQ and chromatographic selectivity is ensured [39,40,41,42,43,44]. A fit-for-purpose approach is therefore appropriate, particularly for pharmacokinetic sampling and exposure assessment [43,44,45].

It is important to acknowledge that single-quadrupole mass spectrometer (QDa) detection operated in SIR mode does not provide structural confirmation based on fragmentation patterns and cannot differentiate isobaric compounds. This represents a limitation compared to LC-MS/MS, particularly in highly complex biological matrices.

However, for the present application, chromatographic separation ensured distinct retention times for all analytes, and no interfering peaks were observed in blank serum samples at the monitored *m*/*z* values. In addition, the achieved LLOQ, precision, and accuracy fall within ranges reported for LC-MS/MS methods for similar compounds [9,10,11,16,20], indicating that the analytical performance is adequate for pharmacokinetic sampling and exposure assessment, where concentrations are well above trace levels. Therefore, the method should be considered complementary to LC-MS/MS rather than a direct replacement, particularly for applications requiring high structural specificity or ultra-trace quantification.

Validation results confirmed the robustness of the method: stability values were above 95%, calibration curves were linear (r > 0.996), precision was below 10%, and accuracy ranged between 95 and 109.20%.

Epirubicin levels at 2 h post-dose (0.125–0.220 µg/mL), olaparib levels at 10 h (1.3–3.0 µg/mL), and ribociclib levels at 2 h (0.547 µg/mL) were consistent with reported pharmacokinetic profiles [23,24,25,26,27,28,29,30,31,32].

The measured concentrations are in agreement with previously reported pharmacokinetic data for these drugs, supporting the reliability of the method for real-sample analysis. Epirubicin concentrations measured 2 h post-dose correspond to the expected post-distribution phase [29]. Olaparib levels at 10 h post-dose fall within the reported steady-state concentration range [30,31], while ribociclib concentrations are consistent with known exposure levels during clinical treatment [25,26,27,28,32].

The therapeutic drug monitoring of targeted anticancer agents is increasingly used for exposure assessment rather than strict dose adjustments [46,47]. In this context, the present assay is suitable for pharmacokinetic studies and provides a proof-of-concept for exposure assessment, while further studies with extended sampling and larger patient cohorts would be required to support adherence evaluation or clinical application. However, due to the absence of a stable isotope-labelled internal standard, the method should not be considered a fully validated therapeutic drug monitoring (TDM) assay for routine clinical decision-making. Its performance approaches that of MS/MS for concentrations well above the LLOQ, although it remains less suitable for ultra-trace quantification or highly standardized TDM applications.

The method provides an alternative for laboratories equipped with single-quadrupole LC-MS systems but without access to tandem mass spectrometry. However, these limitations restrict its application in a fully standardized clinical TDM. Future work will include stable isotope-labelled internal standards to further improve robustness and inter-laboratory comparability

## 5. Conclusions

A chromatographic method with QDa detection was developed and validated for the quantification of epirubicin, olaparib, and ribociclib in human serum using a small sample volume (100 µL). Solid-phase extraction provided efficient sample clean-up and low baseline noise, enabling the sensitive and reproducible detection of all analytes. The analysis of patient samples demonstrated the applicability of the assay in real clinical matrices. Due to its accessibility and the operational simplicity of QDa detection, the method represents a practical alternative for laboratories without LC-MS/MS instrumentation and is suitable for pharmacokinetic sampling, representing a proof-of-concept for exposure assessment, while further studies are required to support routine clinical or adherence-based applications. However, given the absence of an internal standard and confirmatory fragmentation, the assay should be regarded as a research-oriented or supportive clinical tool rather than a fully standardized therapeutic drug monitoring procedure. Future incorporation of a stable isotope-labelled internal standard may further extend its use toward routine clinical settings.

## Figures and Tables

**Table 1:** Stability tests of the analytes (mean ± SD and percentage, *n* = 5).

Analyte/ Target Concentration (ng/mL)	Long-Term Stability	Freeze–Thaw Stability	Room-Temperature Stability
Epirubicin/5	4.77 ± 0.08 (95.40%)	4.76 ± 0.07 (95.20%)	4.76 ± 0.08 (95.20%)
Epirubicin/50	48.01 ± 0.54 (96.02%)	47.85 ± 0.48 (95.70%)	48.05 ± 0.62 (96.10%)
Epirubicin/200	195.04 ± 3.75 (97.52%)	193.08 ± 3.24 (96.54%)	194.20 ± 3.56 (97.10%)
Epirubicin/500	490.25 ± 4.57 (98.05%)	489.05 ± 5.67 (97.81%)	487.80 ± 6.55 (97.56%)
Epirubicin/1000	989.21 ± 8.65 (98.92%)	991.13 ± 9.05 (99.11%)	984.05 ± 10.16 (98.40%)
Olaparib/10	9.52 ± 0.25 (95.22%)	9.59 ± 0.32 (95.91%)	9.62 ± 0.28 (96.23%)
Olaparib/100	96.91 ± 2.24 (96.91%)	97.33 ± 2.48 (97.33%)	96.58 ± 3.05 (96.58%)
Olaparib/1000	973.42 ± 11.25 (97.34%)	969.52 ± 10.89 (96.95%)	976.87 ± 11.75 (97.68%)
Olaparib/2000	1958.41 ± 23.04 (97.92%)	1965.08 ± 27.51 (98.25%)	1955.23 ± 26.35 (97.76%)
Olaparib/5000	4893.52 ± 58.25 (97.87%)	4943.13 ± 62.04 (98.86%)	4945.54 ± 64.84 (98.91%)
Ribociclib/100	95.27 ± 2.57 (95.27%)	96.02 ± 2.21 (96.02%)	96.37 ± 2.64 (96.37%)
Ribociclib/250	241.13 ± 6.23 (96.48%)	242.91 ± 5.94 (97.16%)	242.17 ± 6.58 (96.87%)
Ribociclib/2000	1955.62 ± 15.68 (97.78%)	1938.83 ± 16.36 (96.94%)	1943.02 ± 16.58 (97.15%)
Ribociclib/5000	4869.51 ± 52.36 (97.39%)	4873.57 ± 49.62 (97.47%)	4906.06 ± 58.66 (98.06%)
Ribociclib/10,000	9775.23 ± 104.66 (97.75%)	9885.14 ± 109.33 (98.85%)	9907.72 ± 124.98 (99.07%)

**Table 2:** Precision and accuracy data (mean ± SD, *n* = 5).

Analyte	Target Concentration (ng/mL)	Mean Found Level (ng/mL)		RSD %		Accuracy %	
		Intra-Day	Inter-Day	Intra-Day	Inter-Day	Intra-Day	Inter-Day
Epirubicin	5	4.75 ± 0.19	5.46 ± 0.23	8.25	8.54	95.00	109.20
	50	50.8 ± 1.89	51.15 ± 1.93	7.45	7.57	101.60	102.30
	200	195.80 ± 6.79	196.20 ± 6.91	6.94	7.05	97.90	98.10
	500	496.50 ± 13.80	493.00 ± 13.95	5.56	5.66	99.30	98.60
	1000	994.00 ± 21.86	1014.00 ± 23.37	4.40	4.61	99.40	101.40
Olaparib	10	9.73 ± 0.39	10.15 ± 0.43	8.14	8.56	97.30	101.50
	100	102.50 ± 3.94	100.10 ± 3.88	7.70	7.76	102.50	100.10
	1000	994.00 ± 33.49	976.00 ± 33.28	6.74	6.82	99.40	97.60
	2000	1958.00 ± 52.47	1936.00 ± 52.27	5.36	5.40	97.90	96.80
	5000	4930.00 ± 107	4865.00 ± 109	4.38	4.52	98.60	97.30
Ribociclib	100	95.30 ± 3.78	95.10 ± 3.95	7.95	8.32	95.30	95.10
	250	256.00 ± 8.81	257.25 ± 9.06	6.89	7.05	102.40	102.90
	2000	1946.00 ± 67.62	1940.00 ± 65.76	6.95	6.78	97.30	97.00
	5000	5100.00 ± 146	5135.00 ± 149.9	5.75	5.84	102.00	102.70
	10,000	10,190.00 ± 220	10,210.00 ± 254	4.32	4.98	101.90	102.10

## Data Availability

The data supporting the findings of this study are contained within the article. Additional data related to individual patient samples are not publicly available due to privacy and ethical restrictions but may be provided by the corresponding author upon reasonable request and with permission of the relevant ethics committee.

## Abbreviations

The following abbreviations are used in this manuscript:

SPE–LC–MS	Solid-phase extraction–liquid chromatography–mass spectrometry
MS/MS	Tandem mass spectrometry
PARP	Poly(ADP-ribose)polymerase
DAD	Diode array detector
CDK	Cyclin-dependent kinase
ICH	International Council of Harmonisation
SIR	Selected ion recording
LLOQ	Lowest limit of quantitation
CCs	Calibration curves
QC	Quality control
LLE	Liquid–liquid extraction
TDM	Therapeutic Drug Monitoring
ESI	Electrospray ionization
RSD	Relative Standard Deviation
